# The Surgical Diseases of the Genito-Urinary Tract, Venereal and Sexual Diseases

**Published:** 1906-01

**Authors:** 


THE SURGICAL DISEASES OF THE GENITO-URINARY TRACT, VENEREAL AND SEXUAL DISEASES. A Text-Book for Students and Practitioners. By G. Frank Lydston, M.D. Second Edition. Illustrated—233 Engravings and 7 Colored Plates. F. A. Davis Co., Philadelphia, Publishers.

The work of Lydston is too well known to need an introduction.

Not many changes are made in the second edition except in the illustrations, many excellent ones having been added. The treatment of gonorrhea with the organic silver salts necessitated a revision of the chapter dealing with its treatment. The chapter on Strictures is especially to be commended.

Taken as a whole this is one of our most valuable books upon this branch, and is written with a force and interesting style that can only come after long years of actual experience.

